# Review and standardization of cell phone exposure calculations using the SAM phantom and anatomically correct head models

**DOI:** 10.1186/1475-925X-3-34

**Published:** 2004-10-13

**Authors:** Brian B Beard, Wolfgang Kainz

**Affiliations:** 1 Office of Science and Engineering Laboratories, Center for Devices and Radiological Health, U.S. Food and Drug Administration, Rockville, Maryland, USA

## Abstract

We reviewed articles using computational RF dosimetry to compare the Specific Anthropomorphic Mannequin (SAM) to anatomically correct models of the human head. Published conclusions based on such comparisons have varied widely. We looked for reasons that might cause apparently similar comparisons to produce dissimilar results. We also looked at the information needed to adequately compare the results of computational RF dosimetry studies. We concluded studies were not comparable because of differences in definitions, models, and methodology. Therefore we propose a protocol, developed by an IEEE standards group, as an initial step in alleviating this problem. The protocol calls for a benchmark validation study comparing the SAM phantom to two anatomically correct models of the human head. It also establishes common definitions and reporting requirements that will increase the comparability of all computational RF dosimetry studies of the human head.

## 1. Background

Cell phone safety remains a topic of broad public concern that attracts frequent media attention. This attention is focused on two areas of scientific controversy concerning cell phone safety. The first area is that of non-thermal biological effects. The existence of these effects is an important open question, but it is not the topic of this paper. However, if these effects exist, their manifestation will certainly be related to the amount of RF energy deposited in the tissue – RF dosimetry [1]. The second area of controversy, and the topic of this paper, is that of RF dosimetry, specifically computational RF dosimetry. Simply put, this is a computer simulation that estimates the deposition of RF energy, the specific absorption rate (SAR), in the head of a user. Because live human heads can not be safely instrumented for these measurements, computational RF dosimetry provides the best estimate of SAR in actual human heads. For this same reason, compliance testing is done with phantom heads.

The phantom head that is now the world-wide standard for compliance testing is the Specific Anthropomorphic Mannequin (SAM). SAM was developed by members of IEEE Standards Coordinating Committee 34, SubCommittee 2, Working Group 1 (SCC34/SC2/WG1). This working group was created to develop recommended practices for determining SAR in the head via measurement techniques [2]. SAM has also been adopted by the European Committee for Electrical Standardization (CENELEC) [3], the International Electrotechnical Commission [4], Association of Radio Industries and Businesses [5], and Federal Communications Commission [6].

SAM is a lossless plastic shell and ear spacer. Because current technology does not allow reliable measurement of SAR in small complex structures, like a simulated pinna, SCC34/SC2 chose to use a lossless ear spacer on SAM to maximize the energy reaching the head and minimize measurement uncertainty. SAM's dimensions were taken from the 90^th^-percentile anthropometric data corresponding to the adult male head as tabulated by the US Army [7]. The SAM shell is filled with a homogeneous fluid having the average electrical properties of head tissue at the test frequency.

A primary design goal for SAM was that, "SAM shall produce a conservative SAR for a significant majority of persons during normal use of wireless handsets" [2]. To test whether this goal has been met, investigators have used computational RF dosimetry to compare the SAR in SAM to that in anatomically correct models of the human head. These anatomically correct head models are commonly derived from MRI scans. Each two-dimensional scan must be analyzed to identify individual tissue types. The two-dimensional scans must then be merged into a three-dimensional model that maintains smooth boundaries between tissue types [8,9]. Some investigators have found that SAM underestimates SAR in adults and children by a factor of two or more [10]. Other investigators have found that SAM overestimates SAR in both adults and children [11,12]. These contradictory findings produce confusion on the part of the public and regulatory agencies, and call the validity of computational RF dosimetry into question. While the published results of computational RF dosimetry comparing the SAM to anatomically correct models appear contradictory, a close examination of the work reveals that there are several procedural and reporting problems that may well account for the discrepancies in results.

The groups headed by Gandhi and Kuster are not the only ones pursuing computational RF dosimetry using anatomically correct models of the human head [13-25]. Not all of these studies included SAM but, to various extents, all evidenced the same procedural and reporting problems that make comparison of results difficult.

## 2. Problem areas

### 2.1 Treatment of the pinna

The first, and the most significant of these problems, is the treatment of the external ear (pinna). Specifically, the problem is determining whether the pinna may, or may not, be considered as part of the 1- or 10-gram SAR averaging volumes. When considering SAR averaging volumes the head and the pinna should be viewed as mutually exclusive, in-other-words the pinna is not part of the head but it is attached to the head. Some investigators have chosen to treat the pinna in accordance with IEEE Std C95.1-1999 [26] and the ICNIRP Guidelines [27]. These standards do not consider the pinna to be an extremity. This means the pinna is subject to the same exposure limit, for peak spatial SAR, as the head. Investigators that refer to these standards include pinna tissue in the 1- or 10-gram averaging volumes used to compute SAR in anatomically correct models. Because the pinna is usually the tissue closest to the feed-point of the cell phone antenna the highest point SAR values are usually found in the pinna; consequently, averaging volumes that include pinna tissue will produce higher SAR.

Other investigators have treated the pinna in accordance with draft revision IEEE Std C95.1-200X. This draft standard expands the definition of extremity to include the pinna, which makes the pinna subject to a higher spatial peak SAR, see Table 1. These investigators exclude pinna tissue from their head tissue SAR averaging.

**Table 1:** SAR limits SAR limits from three different standards for extremities and other tissues. These limits are for exposure of the general public in an uncontrolled environment.

	ICNIRP 1998	IEEE C95.1-1999	IEEE C95.1-200X
Extremities	4 W/kg over 10 g	4 W/kg over 10 g	4 W/kg over 10 g
Other tissues	2 W/kg over 10 g	1.6 W/kg over 1 g	2 W/kg over 10 g

When comparing published results it is often difficult, or impossible, to determine whether head tissue SAR values are based on averaging volumes that include or exclude the pinna. In fact, some papers make no mention of how the pinna was treated. Although head tissue SAR is the major focus of attention, papers that consider the pinna as an extremity can not simply ignore its existence, the pinna must still meet the higher spatial peak SAR for extremities.

Another part of the problem dealing with the treatment of the pinna is simply determining what tissue constitutes the pinna. The IEEE defines the pinna as, the largely cartilaginous projecting portion of the outer ear consisting of the helix, lobule, and anti-helix [2]. Unfortunately these anatomical structures vary with each individual and their boundaries are subjective. Consequently, when excluding the pinna some investigators have excluded considerably more or less tissue than others. Because the pinna contains high SAR values, excluding or including tissue near the pinna from the averaging volume, markedly changes the peak spatial 1- or 10-gram average.

### 2.2 Models

The second problem area is the lack of common models. The only computer models that are common to all the computational RF dosimetry studies are the SAM and the Visible Human Male. The anatomic data for the Visible Human Male originated at the National Institutes of Health but many groups and individuals lent a hand in converting it into a computational model. While a few investigators have different models the only ones that can be compared across all the published results are SAM and the Visible Human. This also means that the only repeatable comparison that can be made is between the SAM and the Visible Human. It seems obvious that one can neither prove nor disprove that SAM produces a SAR greater than the maximum local SAR induced in humans for a significant majority of persons during normal use of wireless handsets, when there is only one anatomically correct model available for comparison.

Although not a major problem, it is still true that dielectric properties and names of tissue types in anatomically correct models have varied between investigators.

Of course the head model is only half of any computational RF dosimetry study, the model of the RF source is the other half. The only common source that has been used in several published studies is a dipole [15,28-30]. Simulated cell phones have varied in size, shape, antenna type, antenna length, and sophistication. Like the anatomical head models there are some very realistic models of cell phones in use but they are either proprietary or too expensive for widespread use.

### 2.3 Positioning

**Figure 1:**
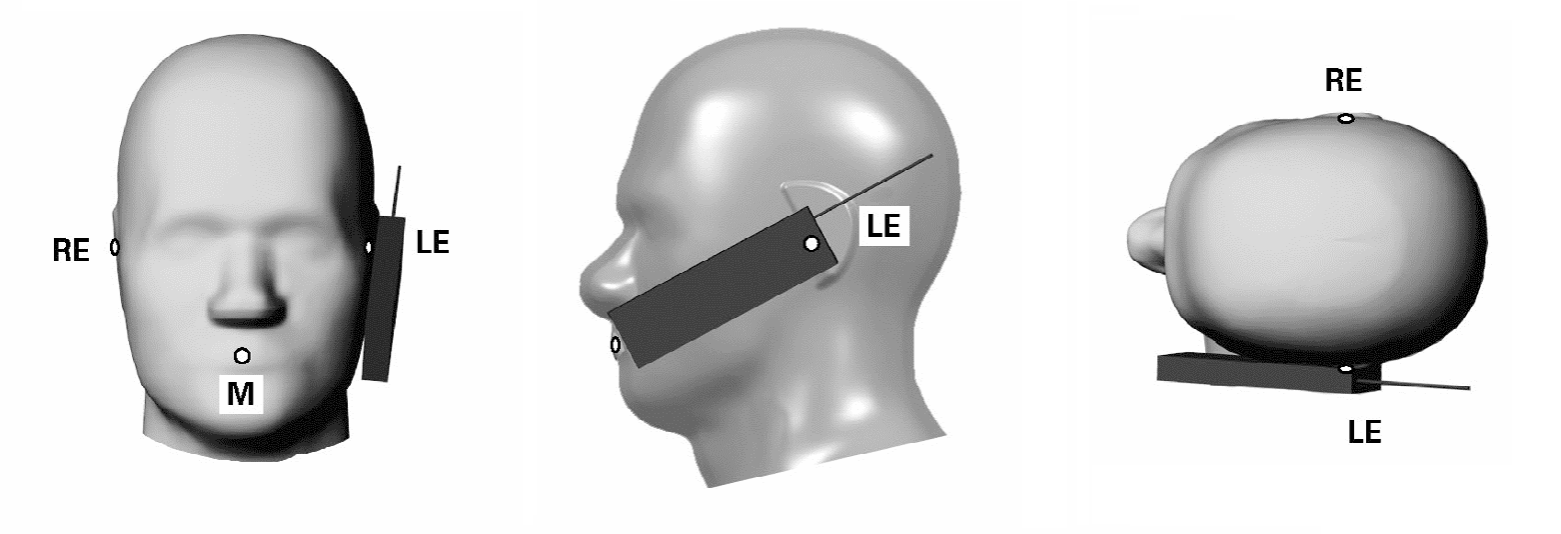
**Touch position. **Specific Anthropomorphic Mannequin with cell phone in touch position on the left side. RE = Right Ear, LE = Left Ear, M = Mouth.

**Figure 2:**
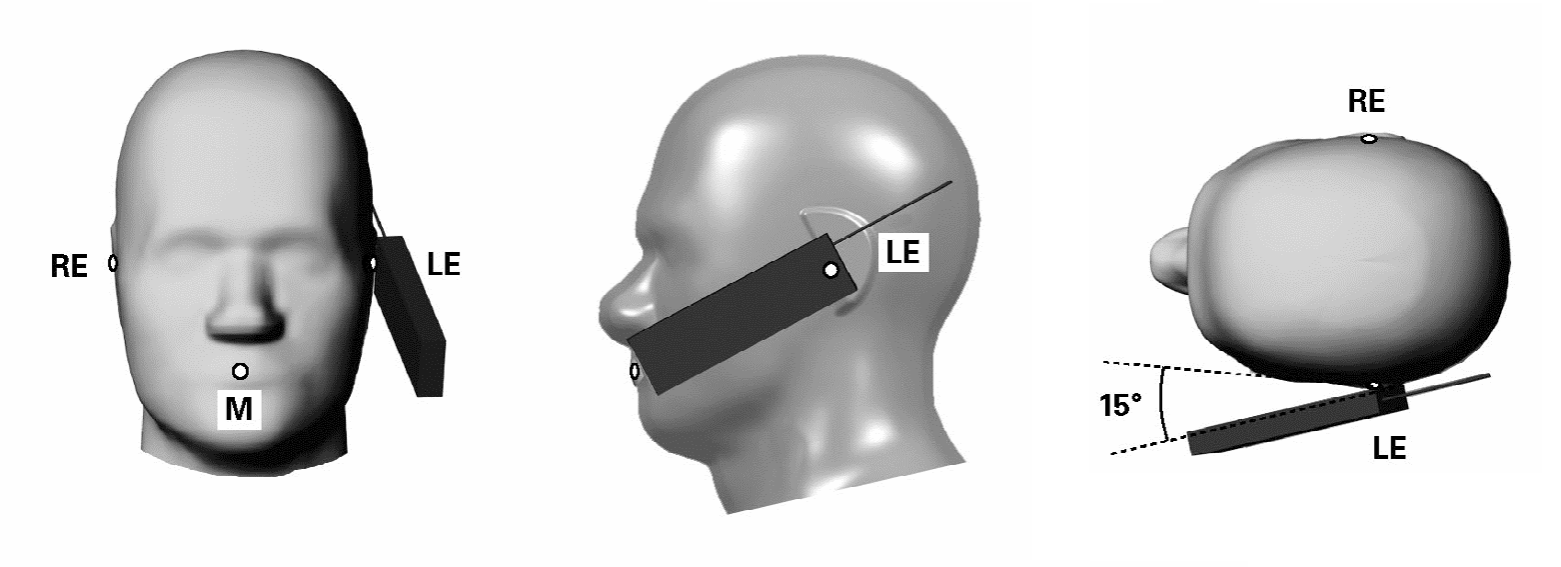
**Tilt position. **Specific Anthropomorphic Mannequin with cell phone in tilted position on the left side. RE = Right Ear, LE = Left Ear, M = Mouth.

The third problem is that of inconsistent positioning of the model cell phone relative to the head model. Simulated SAR in near-field situations is mainly a function of the geometry of the RF current density distribution on the source model and its geometric separation from the lossy head tissue [2]. When the separation distance is small a one or two mm change can significantly alter the observed SAR [30,31]. The CAD files defining SAM show specific reference points and lines used to position cell phones for compliance testing. IEEE Std. 1528 defines two test positions for compliance testing, the touch and tilted position, see Figures 1 and 2 respectively. These positions are routinely used in computational RF dosimetry studies but the anatomical head models do not have defined reference points. These reference points are defined with respect to anatomical features but, as with the definition of the pinna, the interpretation of these anatomical features can vary from investigator to investigator. Consequently, even if two investigators are using the same cell phone and head model, there is no assurance that their positioning of the cell phone relative to the head model is the same.

### 2.4 Finite Difference Time Domain (FDTD) considerations

#### 2.4.1 Rotation artifacts

Usual practice is to align a monopole cell phone antenna with the FDTD grid to avoid the stairstep effect. The head model is then rotated to the correct position relative to the cell phone. After rotation, the voxelized model must be remeshed to align the voxels with the FDTD grid. This is not a trivial task and algorithms to perform remeshing are constantly being improved.

**Figure 3:**
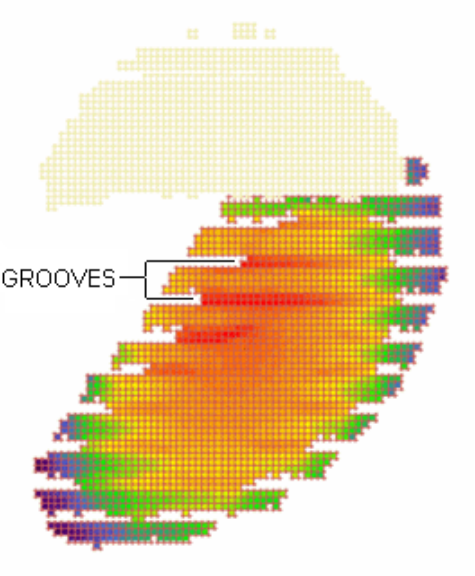
**Artifacts in slice through ear and cheek of SAM. **Slice through ear spacer and cheek of the Specific Anthropomorphic Mannequin (SAM). Two of the many groove artifacts caused by rotation and remeshing are annotated. The upper portion of the figure is the ear spacer which, because it is lossless, has no Specific Absorption Rate (SAR). The lower portion of the figure shows the SAR in the simulated tissue just inside the shell of SAM; red is the highest SAR, violet is the lowest SAR.

The authors have noted some unintended artifacts in voxelized models after remeshing. The first of these is grooving. Figure 3 shows a planar slice through the ear spacer and cheek of the SAM. Note the grooves in what should be a smooth surface. The SAR is zero in the grooves but at the end of the grooves it is higher than in the surrounding voxels due to high E fields within the grooves. These artifacts can distort both the magnitude and location of the peak spatial SAR.

**Figure 4:**
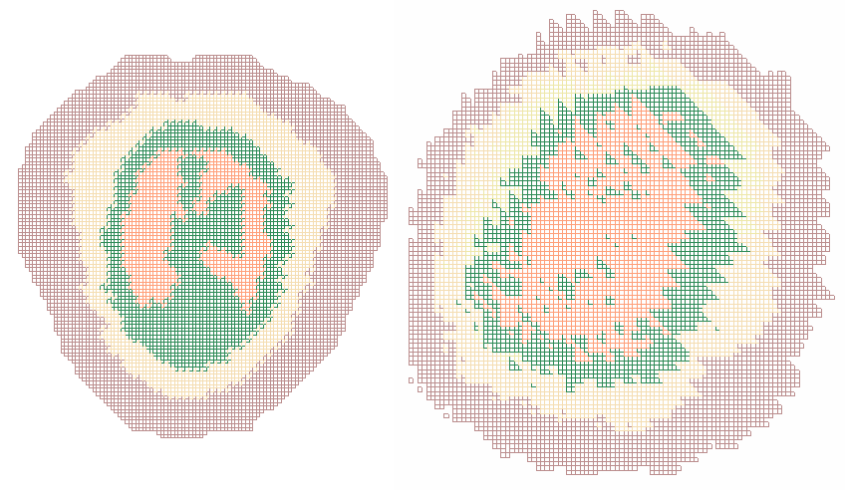
**Artifacts in slice through anatomically correct model. **The image on the left is an XY slice through an unrotated anatomically correct model of a human head. Each color represents a different tissue type. Each tissue type comprises a contiguous region and the boundaries between types are smooth. The image on the right is another XY slice through the same model after rotation around all three axes and remeshing; this is not the same plane represented by the image on the left because that plane is no longer parallel to any of the coordinate axes. In the image on the right tissue types are no longer contiguous regions and the boundaries between types show an unrealistic sawtooth pattern.

The jagged edges caused by grooving are not limited to surface features. Figure 4 shows unrotated and rotated slices through the same anatomic model. The smooth interface between tissue types has been distorted and isolated regions of different tissue types have been created in some locations.

Grooving has not been observed with all FDTD software and even when it has been seen it has not occurred with all models. Researchers should routinely examine their models after rotation to insure grooving is not a problem.

**Figure 5:**
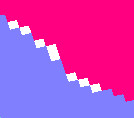
**Empty voxels along tissue boundary. **This image is a close-up of empty voxels caused by rotation and remeshing along a tissue boundary. The white areas are empty voxels along the boundary between the two tissue types indicated by red and blue.

All FDTD programs must, of necessity, perform their calculations on voxelized models. However some programs use CAD models which are only converted to voxelized format after all rotation has been done. These programs avoid most coordinate transformation problem but they are not infallible. They must still convert smoothly undulating biological surfaces into rectilinear voxels. Figure 5 shows empty voxels (air) along a tissue interface where they should not exist.

#### 2.4.2 SAR Calculations

Because the FDTD method calculates the electric fields at the voxel edges, the X, Y and Z-directed power components associated with a voxel are defined in different spatial locations. These components must be combined to calculate SAR in the voxel. There are three possible approaches to calculate the SAR: the 3-, 6-, and 12-field components approaches. The 12-field components approach is the most complicated but it is also the most accurate and the most appropriate from the mathematical point of view [32]. The 12-field components approach correctly places all E field components in the center of the voxel using linear interpolation. Therefore, the power distribution is now defined at the same location as the tissue mass. For these reasons the 12-field components approach is preferred by IEEE 1529 [33]. However, the actual approach used to calculate SAR in the FDTD voxels is usually not reported.

After the SAR in every voxel is determined multiple voxels must be combined to compute the 1- or 10-gram SAR spatial averaging volumes. These normally cubic volumes become difficult to construct at the surface of a model or when the volume is constrained to a particular tissue type. The particular algorithm used to construct these volumes can influence the resultant 1- or 10-gram SAR values. However, the actual algorithm used to construct the spatial averaging volumes is usually not reported.

### 2.5 Reporting results

When the cell phone model is placed next to the SAM or anatomically correct model, it changes the cell phone's antenna feed-point impedance. The antenna feed-point impedance (Z), feed-point current (I) and net input power (P_net_) are related by



Because net power and feed-point current are usually not initial conditions in FDTD simulations, different feed-point impedances will produce different results for net power, feed-point current, and SAR. If different head models produce the same feed-point impedance this would not be a concern; however, several studies [16,17] have shown that the feed-point impedance depends on the head model, the size of the head next to the mobile phone and the mobile phone model itself.

To compare SAM with various anatomic models it is necessary to assume the same cell phone model at the same emission level for all simulations. Typically, for a given simulation, the SAR is normalized by feed-point current or net power. The normalized value is then multiplied by the feed-point current or net power level chosen for comparison. Commonly SAR is compared for net input power levels of 125 mW, 600 mW, or 1 W or for the corresponding feed-point current assuming a 50 ohm feed-point impedance.

Some investigators have chosen to scale their results to net power while others have used feed-point current. Unfortunately the choice of scaling is frequently omitted and the feed-point impedance is almost never reported making it impossible to compare differently scaled results.

## 3. A possible solution

To address the controversy and its underlying problems the Protocol for the Computational Comparison of the SAM Phantom to Anatomically Correct Models of the Human Head was developed by IEEE Standards Coordinating Committee 34, SubCommittee 2, Working Group 2 (SCC34/SC2/WG2). This working group was created to develop recommended practices for determining SAR in the head via computational techniques [33]. This standard is still in draft.

The protocol has two parts; a benchmark validation study; and a set of common definitions, models, and reporting requirements. The benchmark validation study is underway with fifteen participants. All participants should finish the study by mid-2004 and the results should be published by early 2005. Hopefully the common definitions, models, and reporting requirements will be used in future investigations making comparison of results easier.

### 3.1 Treatment of the pinna

The protocol asks all participants to report peak spatial SAR for averaging volumes that both include and exclude the pinna. The voxels comprising the pinna in the provided anatomic models are flagged so all participants will conform to one definition of the pinna. The pinna voxels are flagged by prefixing the standard tissue type with pinna-; such as pinna-skin, pinna-cartilage, and pinna-fat. The electrical properties of the flagged pinna voxels are unchanged. The IEEE Std 1528 definition for the pinna was followed and the choice of each flagged voxel was confirmed by an Ear-Nose-Throat surgeon.

### 3.2 Models

The Benchmark Validation Study calls for each participant to run twelve simulations: three head models, at two frequencies (835 and 1900 MHz), and in two cell phone positions (touch and tilted). The models are SAM, the Visible Human, and a seven year old Japanese male [16]. Each model is provided as a voxel file with an ASCII header file.

For the two anatomically correct models, the tissue names and properties in the header file were made consistent with the definitions found on the Italian National Research Council, Institute for Applied Physics web site [34].

Although they are not part of the benchmark validation study, SCC34/SC2/WG2 plans on releasing several new anatomically correct models in the next few months to expand the population of models available for study.

**Figure 6:**
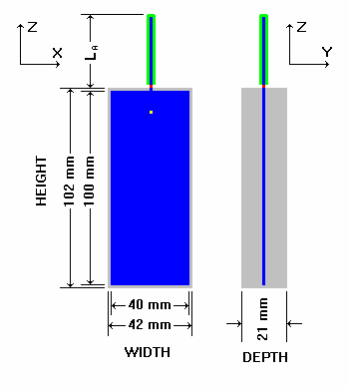
**Generic cell phone. **The Generic cell phone designed for the intercomparison protocol. Blue = perfect electrical conductor, gray = plastic insulator, green = rubber insulator, red = antenna feed-point voltage source, yellow = acoustic output.

A generic cell phone is described for use in all benchmark validation studies, see Figure 6. The length of the antenna is 71 mm for 835 MHz and 36 mm for 1900 MHz. Because the cell phone and SAM are symmetric, and the anatomically correct models are approximately symmetric, SCC34/SC2/WG2 chose to do all simulations with the phone on the right hand side of the head.

### 3.3 Positioning

The reference points, necessary for positioning the cell phone relative to the anatomically correct model, are also contained in the header file for each model.

**Figure 7:**
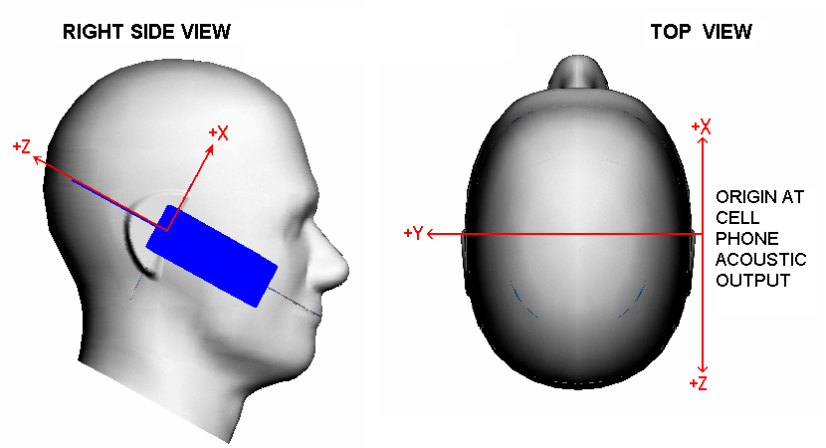
**Coordinate system. **The left image shows the cell phone referenced coordinate system as seen from the right side of the Specific Anthropomorphic Mannequin (SAM). The right image shows the coordinate system as seen from the top of the SAM. The SAM Ear Reference Points, left and right, are where the Y axis intercepts the surface of the mannequin.

To aid comparison of results from all the participants, a common coordinate system was defined with origin at the acoustic output of the cell phone, see Figure 7. The participants are asked to report the following positioning data for all simulations: the distance between the antenna feed-point and the nearest tissue voxel, the coordinates of the Ear Reference Point (ERP), and the direction cosines (as a rotation matrix) for the coordinate transformation of the head models for touch and tilted positions. As defined in IEEE Std 1528, the ERP is 15 mm posterior to the ear canal in the plane passing through the mouth and both ear canals.

### 3.4 FDTD considerations

The FDTD technique is called for by the P1529 draft [33]. FDTD was chosen because it is stable and accurate, doesn't require enormous computational resources and can handle complex geometries.

The participants are asked to report the following FDTD data for all simulations: The boundary conditions used and the minimum distance between the model and the boundary of the computational space. The time step size and the number of time steps used. The grid (voxel) size and whether the grid was homogeneous or graded. To calculate the SAR in each voxel the protocol recommends the 12-field components approach. All 1- or 10-gram spatial averaging volumes are to be constructed in accordance with IEEE C95.3, Annex E.

### 3.5 Reporting

The participants are asked to report the following SAR data for all simulations: The peak spatial SAR, both 1 g and 10 g averages, for all tissue (head plus pinna), head only, and pinna only averaging volumes and the location of the averaging cubes. The peak point value SAR and its location. A color coded SAR distribution for both 1 g and 10 g averages, in the ZY plane.

#### 3.5.1 Scaling reported results

For a realistic study it would be ideal to simulate the real world situation. The question that remains is: "Does a real-world cell phone keep the power or the feed-point current constant when placed next to different human beings with different head shapes and head sizes"? Unfortunately there is not a definitive answer to this question. The behavior of a mobile phone depends on the system design and the power amplifier circuits. A detailed discussion for real-world mobile telephones has to be addressed by future projects with more realistic mobile phones models for numerical simulations.

For now it is important to scale the calculated SAR values to net input power and feed-point current and to present both results. The behavior of a real-world mobile phone is within the SAR range of scaling to the net input power and scaling to the feed-point current. For human health and safety considerations a worst case approach is desirable. Until further knowledge on the behavior of a real-world cell phone is available, the scaling producing the worst case result (largest SAR value) must be taken into account.

## 4. Conclusion

The current version of IEEE Std C95.1 [26] does not classify the pinna as an extremity making it subject to the basic SAR exposure limitation of 1.6 W/kg over 1 g. However the much anticipated 200X revision of C95.1 will reclassify the pinna as an extremity raising its SAR exposure limit to 4 W/kg over 10 g. Confusion over the inclusion or exclusion of the pinna in the SAR averaging volume will continue until the IEEE officially releases C95.1-200X. The IEEE should release C95.1-200X as soon as practical, and if this can not be done in a reasonably short time, a supplement should be published clarifying the new status of the pinna as an extremity.

Investigators should inspect all models after rotation to be sure they are free of artifacts caused by meshing along the new coordinate axes. If necessary, artifacts should be manually corrected before running the simulation. Blindly accepting the output of meshing algorithms can lead to errors. All relevant data and assumptions for the computational RF dosimetry study, as discussed in section 2 "Problem areas", must be reported in such detail that the reader is able to compare the results to other studies. The names and electrical properties for all anatomically correct models should comply with those shown on the Italian National Research Council, Institute for Applied Physics web site.

To facilitate broad based comparisons, new anatomically correct models should be placed in the public domain or made available for a modest fee. The number of anatomically correct models suitable for electromagnetic modelling and widely available for comparison to the SAM is still low. Because the SAM is intended to represent a significant majority of persons during normal use of wireless handsets, comparison to a large variety of anatomically correct models is desirable. It is the hope of IEEE SCC34/SC2/WG2 that consistent results in the benchmark validation will show that, by adhering to some common definitions and procedures, FDTD studies from different investigators using different anatomically correct models are comparable.

## Authors' contributions

BB, past chairman of IEEE SCC34/SC2/WG2, drafted the **Protocol for the Computational Comparison of the SAM Phantom to Anatomically Correct Models of the Human Head **and this manuscript. WK, present chairman of IEEE SCC34/SC2/WG2, wrote approximately 25% of the manuscript, developed and simulated the phone model and supplied several of the figures. Both authors read and approved the final manuscript.

## References

[B1] Lin JC (2002). Cellular Mobile Telephones and Children. IEEE Antennas and Propagation Magazine.

[B2] IEEE Std 1528-2003 Recommended Practice for Determining the Peak Spatial-Average Specific Absorption Rate (SAR) in the Human Head from Wireless Communications Devices – Measurement Techniques. Institute of Electrical and Electronics Engineers, New York.

[B3] EN 50361 (2001). Basic Standard for the Measurement of Specific Absorption Rate Related to Exposure to Electromagnetic Fields from Mobile Phones (300 MHz – 3 GHz). European Committee for Electrical Standardization (CENELEC), Brussels.

[B4] IEC 62209 Procedure to measure the Specific Absorption Rate (SAR) in the frequency range of 300 MHz to 3 GHz – Part 1: hand-held mobile wireless communication devices. International Electrotechnical Commission, committee draft for vote.

[B5] ARIB STD-T56 (2002). Specific Absorption Rate (SAR) Estimation for Cellular Phone. Association of Radio Industries and Businesses.

[B6] Supplement C to OET Bulletin 65 (Edition 9701) (1997). Evaluating Compliance with FCC Guidelines for Human Exposure to Radio Frequency Electromagnetic Fields. Federal Communications Commission (FCC), Washington, DC.

[B7] Gordon CC, Churchill T, Clauser CE, Bradtmiller B, McConville JT, Tebbetts I, Walker RA (1989). 1988 Anthropometric Survey of U.S. Army Personnel: Methods and Summary Statistics, Technical Report NATICK/TR-89/044. US Army Natick Research, Development and Engineering Center, Natick, Massachusetts.

[B8] Mazzurana M, Sandrini L, Vaccari A, Malacarne C, Cristoforetti L, Pontalti RA (2003). Semi-automatic method for developing an anthropomorphic numerical model of dielectric anatomy by MRI. Physics in Medicine and Biology.

[B9] Gjonaj E, Bartsch M, Clemens M, Schupp S, Weiland T (2002). High-resolution human anatomy models for advanced electromagnetic field computations. IEEE Transactions on Magnetics.

[B10] Gandhi OP, Kang G (2002). Some present problems and a proposed experimental phantom for SAR compliance testing of cellular telephones at 835 and 1900 MHz. Physics in Medicine and Biology.

[B11] Kuster N, Christ A, Chavannes N, Nikoloski N, Frolich J Human Head Phantoms for Compliance and Communication Performance Testing of Mobile Telecommunication Equipment at 900 MHz. 2002 Interim International Symposium on Antennas and Propagation, Yokosuka Reserach Park, Japan.

[B12] Christ A, Chavannes N, Pokovic K, Gerber H, Kuster N (2000). Numerical and Experimental Comparison of Human Head Models for SAR Assessment. Proceedings of the Millennium Workshop on Biological Effects of Electromagnetic Fields, Heraklion, Kreta, Greece.

[B13] Lee A, Choi H, Lee H, Pack J (2002). Human head size and SAR characteristics for handset exposure. ETRI Journal.

[B14] Dimbylow PJ, Mann SM (1994). SAR calculations in an anatomically realistic model of the head for mobile communications transceivers at 900 MHz and 1.8 GHz. Physics in Medicine and Biology.

[B15] Van de Kamer JB, Lagendijk JJW (2002). Computation of high-resolution SAR distributions in a head due to a radiating dipole antenna representing a hand-held mobile phone. Physics in Medicine and Biology.

[B16] Wang J, Fujiwara O (2003). Comparison and evaluation of electromagnetic absorption characteristics in realistic human head models of adult and children for 900-MHz mobile telephones. IEEE Transactions on Microwave Theory and Techniques.

[B17] Okoiewski M, Stuckly MA (1996). A study of the handset antenna and human interaction. IEEE Trans. Microwave Theory Tech.

[B18] Hombach V, Meier K, Burkhardt M, Kühn E, Kuster N (1996). The dependence of EM energy absorption on human head modeling at 900 MHz. IEEE Transactions on Microwave Theory and Techniques.

[B19] Bernardi P, Cavagnaro M, Pisa S (1996). Evaluation of the SAR distribution in the human head for cellular phones used in a partially closed environment. IEEE Transactions of Electromagnetic Compatibility.

[B20] Wiart J, Dale C, Bosisio AV, Le Cornec A (2000). Analysis of the influence of the power control and discontinuous transmission on RF exposure with GSM mobile phones. IEEE Transactions on Electromagnetic Compatibility.

[B21] Luebbers R, Baurle R FDTD Predictions of Electromagnetic Field in and near Human Bodies Using Visible Human Project Anatomical Scans. IEEE AP-S International Symposium and URSI Radio Science Meeting, Baltimore, MD.

[B22] Martinez-Burdalo M, Martin A, Anguiano M, Villar R (2004). Comparison of FDTD-calculated specific absorption rate in adults and children when using a mobile phone at 900 and 1800 MHz. Physics in Medicine and Biology.

[B23] Gandhi OP, Lazzi G, Furse CM (1996). Electromagnetic Absorption in the Human Head and Neck for Mobile Telephones at 835 and 1900 MHz.. IEEE Trans Microwave Theory and Techniques.

[B24] Hadjem A, Lautru D, Dale C, Wong M, Fouad-Hanna V, Wiart J (2004). Comparison of Specific Absorption Rate (SAR) Induced in Child-Sized and Adult Heads Using a Dual Band Mobile Phone. Proceedings on IEEE MTT-S International Microwave Symposium IMS.

[B25] Mochizuki S, Watanabe S, Taki M, Yamanaka Y, Shirai H (2004). Size of Head Phantoms for Standard Measurements of SAR Due to Wireless Communication Devices. Electronics and Communications in Japan, Part 1.

[B26] IEEE Std C95.1-1999 IEEE Standard for Safety Levels with Respect to Human Exposure to Radio Frequency Electromagnetic Fields, 3 kHz to 300 GHz. Copyright 1992 by the Institute of Electrical and Electronics Engineers (IEEE), Inc, New York, NY.

[B27] (1998). ICNIRP (International Commission on Non-Ionizing Radiation Protection), Guidelines for Limiting Exposure to Time-Varying Electric, Magnetic and Electromagnetic Fields (Up to 300 GHz). Health Physics.

[B28] Anderson V (2003). Comparisons of peak SAR levels in concentric sphere head models of children and adults for irradiation by a dipole at 900 MHz. Physics in Medicine and Biology.

[B29] Kanda M, Balzano Q, Russo P, Faraone A, Bit-Babik G (2002). Effects of ear-connection modeling on the electromagnetic-energy absorption in a human head phantom exposed to a dipole antenna field at 835 MHz. IEEE Transactions on Electromagnetic Compatibility.

[B30] Schönborn F, Burkhardt M, Kuster N (1998). Differences in energy absorption between heads of adults and children in the near field of sources. Health Physics.

[B31] Kuster N, Balzano Q (1992). Energy absorption mechanism by biological bodies in the near field of dipole antennas above 300 MHz. IEEE Transactions on Vehicular Technology.

[B32] Caputa K, Okoniewski M, Stuchly MA (1999). An Algorithm for Computations of the Power Deposition in Human Tissue. IEEE Antennas and Propagation Magazine.

[B33] IEEE 1529 Recommended Practice for Determining the Spatial-Peak Specific Absorption Rate (SAR) Associated with the Use of Wireless Handsets – Computational Techniques. Institute of Electrical and Electronics Engineers, New York, draft standard.

[B34] Dielectric Properties of Body Tissue in the frequency range 10 Hz – 100 GHz. Italian National Research Council, Institute for Applied Physics, Florence, Italy.

